# Acetylation of Steroidogenic Acute Regulatory Protein Sensitizes 17β-Estradiol Regulation in Hormone-Sensitive Breast Cancer Cells

**DOI:** 10.3390/ijms25168732

**Published:** 2024-08-10

**Authors:** Pulak R. Manna, Deborah Molehin, Ahsen U. Ahmed, Shengping Yang, P. Hemachandra Reddy

**Affiliations:** 1 Department of Internal Medicine, School of Medicine, Texas Tech University Health Sciences Center, Lubbock, TX 79430, USA; hemachandra.reddy@ttuhsc.edu; 2 College of Veterinary Medicine, Midwestern University, Glendale, AZ 85308, USA; dmoleh@midwestern.edu; 3 Comprehensive Cancer Center, University of California Davis, Sacramento, CA 95817, USA; ahsen.ahmeda@ttuhsc.edu; 4 Department of Biostatistics, Pennington Biomedical Research Center, Louisiana State University, Baton Rouge, LA 70808, USA; shengping.yang@pbrc.edu; 5 Neurology, Departments of School of Medicine, Texas Tech University Health Sciences Center, Lubbock, TX 79430, USA; 6 Public Health Department of Graduate School of Biomedical Sciences, Texas Tech University Health Sciences Center, Lubbock, TX 79430, USA; 7 Department of Pharmacology and Neuroscience, Texas Tech University Health Sciences Center, Lubbock, TX 79430, USA; 8 Nutritional Sciences Department, College of Human Sciences, Texas Tech University, Lubbock, TX 79409, USA

**Keywords:** breast cancer, StAR, acetylation, E2 biosynthesis, HDAC inhibitors, therapeutics

## Abstract

An imbalance in estrogen signaling is a critical event in breast tumorigenesis. The majority of breast cancers (BCs) are hormone-sensitive; they majorly express the estrogen receptor (ER+) and are activated by 17β-estradiol (E2). The steroidogenic acute regulatory protein (StAR) mediates the rate-limiting step in steroid biosynthesis. The dysregulation of the epigenetic machinery, modulating E2 levels, is a primary occurrence for promoting breast tumorigenesis. StAR expression, concomitant with E2 synthesis, was reported to be aberrantly high in human and mouse hormone-dependent BC cells compared with their non-cancerous counterparts. However, the mechanism of action of StAR remains poorly understood. We discovered StAR as an acetylated protein and have identified a number of lysine (K) residues that are putatively acetylated in malignant and non-malignant breast cells, using LC-MS/MS (liquid chromatography–tandem mass spectrometry), suggesting they differently influence E2 synthesis in mammary tissue. The treatment of hormone-sensitive MCF7 cells with a variety of histone deacetylase inhibitors (HDACIs), at therapeutically and clinically relevant doses, identified a few additional StAR acetylated lysine residues. Among a total of fourteen StAR acetylomes undergoing acetylation and deacetylation, K111 and K253 were frequently recognized either endogenously or in response to HDACIs. Site-directed mutagenesis studies of these two StAR acetylomes, pertaining to K111Q and K253Q acetylation mimetic states, resulted in increases in E2 levels in ER+ MCF7 and triple negative MB-231 BC cells, compared with their values seen with human StAR. Conversely, these cells carrying K111R and K253R deacetylation mutants diminished E2 biosynthesis. These findings provide novel and mechanistic insights into intra-tumoral E2 regulation by elucidating the functional importance of this uncovered StAR post-translational modification (PTM), involving acetylation and deacetylation events, underscoring the potential of StAR as a therapeutic target for hormone-sensitive BC.

## 1. Introduction

Breast cancer (BC) is the second greatest cause of cancer-related death among women worldwide. In the United States, an estimated 310,720 new cases (32%, the highest among all cancers) and 42,250 deaths with BCs are projected to occur during 2024 [1]. BCs are broadly divided into two categories, i.e., hormone-receptor positive (HR+) and hormone-receptor negative (HR−), based on the receptors they express on their cell surfaces. While HR+ BCs express ER (ER+), especially *ESR1*, progesterone receptor (PR+), and/or human epidermal growth factor receptor 2 (HER2+), HR- or triple negative BC (TNBC) does not express these receptors. Notably, hormone-sensitive BC is primarily triggered by estrogens, particularly E2, which are produced from diverse sources, including extra-ovarian sites and locally within malignant breast epithelial cells [2,3,4,5]. Consequently, cancerous breast tissues express high levels of aromatase (encoded by the *CYP19A1* gene), the enzyme that plays an indispensable role in estrogen/E2 biosynthesis from androgens [6,7,8,9]. However, the expression of this key enzyme is surprisingly high, not only in malignant but also in non-malignant breast tissue [10,11]. While these circumstances concern the diagnostic potential of aromatase in BCs, they also suggest the involvement of additional factor(s) in E2 synthesis in mammary tissue. It is noteworthy, however, that, despite the effectiveness of aromatase inhibitors (AIs) in BC treatment in post-menopausal women, AIs develop endocrine resistance modulating cancer death [12,13,14]. As such, an improved understanding of the molecular underpinnings involving appropriate diagnosis and treatment is the key for combating this devastating disease.

An overwhelming amount of evidence indicates that disruption of the steroidogenic machinery, involving androgen and estrogen biosynthesis, has been associated with the pathogenesis of hormone-sensitive BC and other cancers [4,8,15]. Notably, cholesterol is the precursor for all steroid hormones, including E2, in which the StAR protein (also called STARD1) primarily regulates steroid biosynthesis by controlling the intra-mitochondrial transport of cholesterol [16,17,18,19]. We reported that StAR expression, along with E2 synthesis, is aberrantly high not only in human hormone-dependent BC cells, but also in transgenic mouse models of spontaneous breast tumors, in comparison with little to no expression of StAR in their non-cancerous counterparts or TNBC [4,11,20]. While these findings indicate that StAR acts as a tumor promoter in ER+ BC, its differential expression in malignant and non-malignant breast tissues designates this cholesterol transporter as a new diagnostic marker. Studies have demonstrated that the tissue-specific regulation of steroid biosynthesis, impacting various physiological and pathophysiological activities, is modulated by events/signaling that enhance the translation, transcription, or activity of StAR through endocrine, autocrine, or paracrine mechanisms [21,22,23]. Importantly, while the posttranslational modification (PTM) of StAR, involving phosphorylation, has been shown to enhance its biological activity for optimal steroid biosynthesis, mutations in the StAR gene severely affect steroid biosynthesis [17,24,25].

Acetylation is a highly dynamic PTM that integrates the functional diversity of the proteome by modulating the epigenetic landscape and cell signaling networks [26,27,28]. Reciprocally, acetylation has been shown to influence the activity and function of many proteins in cancer cells [8,10,11]. Along these lines, we uncovered a novel PTM of StAR involving acetylation, and identified several lysine residues, undergoing acetylation and deacetylation, in cancerous and non-cancerous breast cells, using LC-MS/MS (liquid chromatography–tandem mass spectrometry) [11]. Even so, the functional importance of various StAR acetylomes to E2 synthesis, especially in mammary tissue, remains elusive. While histone acetyltransferase (HATs) catalyzes its acetylation, histone deacetylases (HDACs) remove this reversible and dynamic process [29,30]. HDACs are a family of epigenetic enzymes (18 members, in four classes, encoded in the human genome), which regulate numerous cellular processes, including chromatin remodeling and genomic stability through acetylation and deacetylation of core histones [4,31,32]. Alternatively, the dysregulation of HDACs is a fundamental event for the progression of carcinogenesis in breast and other tissues [4,31,33,34]; as a consequence, HDAC inhibitors (HDACIs) have received wide attention for various cancer therapies [9,13,15,16]. Moreover, HDACIs have multiple targets in cancer cells and display many favorable outcomes, including cell cycle arrest, anti-proliferation, apoptosis, and senescence [4,11,35,36,37,38]. In accordance with this, we reported that a variety of HDACIs, at therapeutically relevant doses, alter StAR acetylation patterns and suppress E2 accumulation in human and mouse hormone-sensitive BC cells [11,20].

## 2. Results

### 2.1. Acetylation of StAR Lysine Residues in Cancerous and Non-Cancerous Breast Cells by LC-MS/MS

The hypothesis, that the differential expression of StAR concomitant with E2 levels in malignant and non-malignant breast cells involves the acetylation of this steroid hormone regulator, was assessed using LC-MS/MS. Utilizing human hormone-dependent MCF7, hormone-independent MB-231 (TNBC), and non-cancerous MCF12F breast cell lines, we identified eight constitutive lysine residues that are acetylated in the StAR protein at positions K7, K97, K98, K111, K118, K155, K238, and K253 (Table 1). This novel PTM of StAR was verified by identifying four acetylated lysine residues (K98, K107, K111, and K118), using LC-MS/MS, in human classical steroidogenic H295R adrenocortical cells, suggesting the uniqueness of this uncovered modification in the regulation of steroid hormones.

### 2.2. Effects of a Variety of HDACIs on StAR Acetylation Patterns in MCF7 Cells

Acetylation is a reversible PTM that regulates the function of histone and non-histone proteins involved in tumorigenesis [10,39]. We reported that a number of HDACIs impact the expression and activity of StAR on E2 biosynthesis in human and mouse hormone-sensitive BC cells [11,20]. To understand the mechanistic events associated with HDACI-treated StAR function, a number of acetylated lysine residues in the StAR protein were identified in response to four different HDACIs (Table 2). Specifically, the treatment of MCF7 cells with two United States Food and Drug Administration (FDA)-approved HDACIs, i.e., Panobinostat (10 nM, targets classes I, II, and IV HDACs), and suberoylanilide hydroxamic acid (SAHA)/vorinostat (1 μM, targets HDAC1 and 2) at therapeutically relevant doses identified four additional acetylated StAR lysine residues at K107, K152, K162, and K248 positions using LC-MS/MS (Table 2). Simultaneously, two clinical-phase trial HDACIs and SIRT (Sirtuin) inhibitors, inhibitor IV (IV, 1 μM; affects SIRT1/2) and inhibitor VII (VII, 1 μM, targets SIRT7), at preclinical doses, identified two additional StAR acetylated lysine residues at K21 and K213.

### 2.3. Assessment of Acetylated StAR Lysine Residues in Cancerous and Non-Cancerous Breast Cells

**Figure 1:**
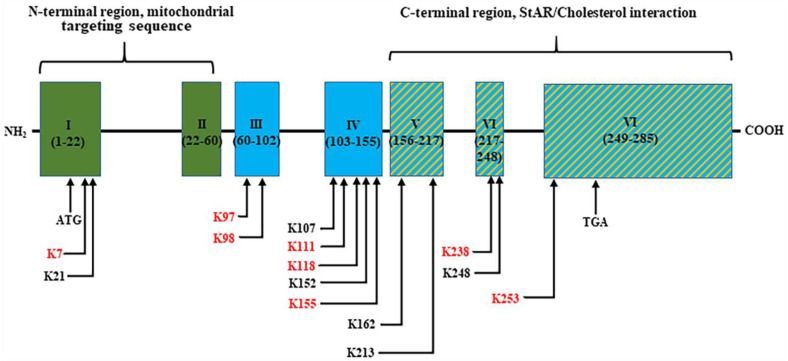
A schematic representation of intron and exon structures (numbered as I–VI) of the *StAR* gene illustrating approximate positions of the identified acetylated lysine residues. Both the NH_2_-terminal mitochondrial targeting sequence and COOH-terminal StAR/cholesterol interacting regions are depicted. Acetylated StAR lysine residues, highlighted in red and black, are identified endogenously and in response to HDACIs, respectively, using LC-MS/MS.

The identification of a total of fourteen StAR acetylated lysine residues, under endogenous and various HDACI-treated conditions, was found to be highly conserved across different species (Figure 1). Thus, lysine residues that undergo acetylation and deacetylation states play discrete roles in the biological activity of StAR in E2 biosynthesis. Notably, among these StAR acetylomes, K111 and K253 were frequently recognized either endogenously or in response to certain HDACIs in MCF7 cells using LC-MS/MS. Furthermore, K253 was located at exon 7, a COOH-terminal helix that interacts with the outer mitochondrial membrane, suggesting that this acetylated StAR lysine residue facilitates the optimal transport of cholesterol for E2 biosynthesis.

### 2.4. Structure of the Human StAR Protein along with K → Q and K → R Substitutions at K111 and K253 Positions

**Figure 2:**
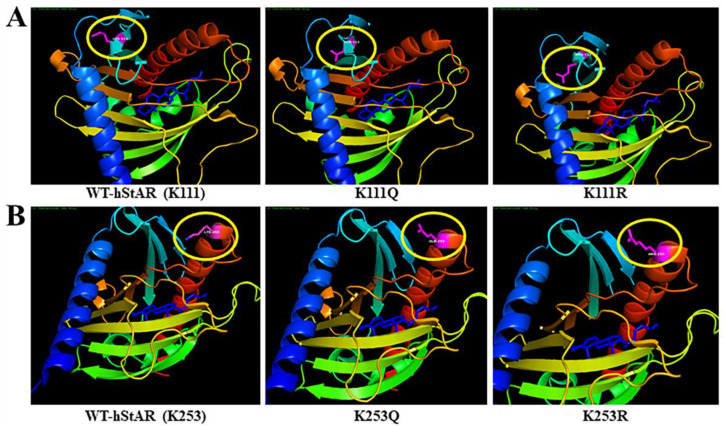
A schematic representation of WT-hStAR protein structure containing K111 (**A**) and K253 (**B**) residues using the PyMOL Molecular Graphics System. Molecular visualizations of the structure of WT-hStAR containing K111 and K253 (**left panels**), along with K → Q and K → R substitutions at K111Q and K253Q (**middle panels**) and K111R and K253R (**right panels**), are depicted in magenta with yellow oval shapes. NH_2_-terminal α-helix (amino acid, aa69–92) is outlined in blue that is followed by anti-parallel β-sheet in cyan (aa97–101, 107–112, and 118–126 interspaced by β-hairpin regions). This leads to two α-helixes separated by short loop structures in green (aa129–138 and 140–144) and mixed beta sheets (aa153–159 and 164–171), which are followed by anti-parallel beta sheets connected by loops in yellow (aa182–192 and 197–199) and in orange (aa202–203; 216–217; 219–220; 224–230; 233–243; 245–246), with the COOH-terminal α-helix in red (aa252–275). The cholesterol structure within the cavity of the StAR protein is delineated in dark blue.

The human StAR protein structure was downloaded from the RCSB (Research Collaboratory for Structural Bioinformatics) Protein Data Bank database (https://www.rcsb.org/structure/3p0l; accessed on 1 March 2023) [40,41]. Three-dimensional structures of human wild-type (WT) StAR (WT-hStAR), comprising K111 and K253, along with K → Q (glutamine) and K → R (arginine) substitutions involving K111Q, K111R, K253Q, and K253R modifications, are depicted in Figure 2. We chose both Q and R residues, because they are similar in length to K, in which lysine acetylation neutralizes the positive charge, Q is neutral, and R maintains its charge. The original positions of WT-hStAR containing K111 and K253 (left panels), and their modifications at K → Q (middle panels) and K → R (right panels) mutants, generated by the PyMOL (Version 2.2.3_0) Molecular Graphics System (Schrodinger, New York, NY, USA), were highlighted with yellow oval shapes. The structural modifications with K → Q and K → R mutants, involving K111 and K253, are expected to differently influence the biological activity of StAR in E2 biosynthesis.

### 2.5. Functional Assessment of K111 and K253 Acetylation and Deacetylation Mutants on E2 Levels in ER+ MCF7 and TNBC MB-231 Cells

**Figure 3:**
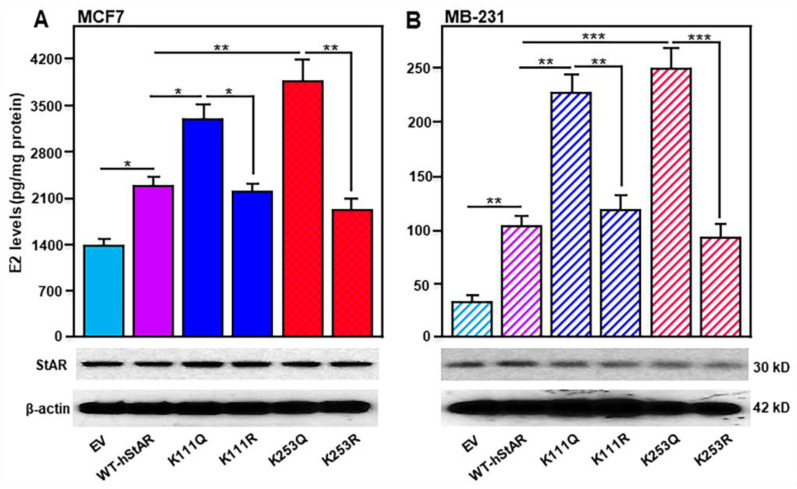
Assessment of WT-hStAR, K111Q, K111R, K253Q, and K253R in E2 levels in MCF7 (**A**) and MB-231 (**B**) cells. These cells were transfected with EV, WT-hStAR, and different K → Q and K → R mutants, as specified, by Lipofectamine 3000 reagent. Following 48 h of transfection, culture media from different groups were analyzed for E2 levels and expressed as pg/mg protein from three to four independent experiments. Simultaneously, cells were subjected to whole cell extract preparation and representative immunoblots illustrate StAR protein expression using 70 μg of total protein. β-actin expression was assessed as loading controls in both panels. Note two different scales in Y-axes for MCF7 (**A**) and MB-231 (**B**) cells. *, *p* < 0.05; **, *p* < 0.01, ***, *p* < 0.001 vs. EV or other groups, as indicated.

Among a total of fourteen acetylated lysine residues in the StAR protein, two at positions K111 and K253 were frequently recognized either endogenously or in response to HDACIs. Thus, the effects of these two StAR acetylomes on E2 synthesis were assessed in conjunction with K → Q and K → R, and acetylation and deacetylation mimetic conditions, respectively, by generating point mutations with site-directed mutagenesis [8,10]. The results presented in Figure 3 reveal that MCF7 (Figure 3A) and MB-231 (Figure 3B) cells overexpressing WT-hStAR resulted in significant increases (*p* < 0.05) in E2 levels over the values seen with respective EV (empty vector, pcDNA3) controls. While both K111Q and K253Q acetylation mutants enhanced E2 synthesis between 52 and 67% compared with WT-hStAR in MCF7 and MB-231 cells, K111R and K253R deacetylation mutants suppressed (*p* < 0.05) E2 levels. These opposing effects in E2 biosynthesis, especially decreasing with K111R and K253R mutants to WT-hStAR, could be influenced by other lysine residues identified in the StAR protein. However, alterations in E2 levels in different groups were not associated with changes in StAR protein expression (Figure 3A,B, bottom panels), which agree with previous studies [8,10]. Additionally, StAR protein expression was found to be relatively higher in MCF7 cells than those of MB-231 and correlated with E2 levels [11,20]. The magnitude of responses with K → Q and K → R mutants in E2 synthesis was noticeably higher in MB-231 cells than those of MCF7 cells, which could be due to an endogenously lower expression of StAR in TNBC cells. Therefore, acetylated StAR lysine residues, especially at K111 and K253, were capable of enhancing E2 accumulation for triggering breast tumorigenesis. Conversely, K111R and K253R deacetylation mutants diminished E2 levels in both MCF7 and MB-231 cells. It is plausible that StAR acetylation and deacetylation events differently modulate E2 biosynthesis for sustaining diverse activities in mammary tissue.

## 3. Discussion

The identification of mass spectrometry has been a powerful tool for various PTM mapping of proteins and/or peptides that are altered in both physiological and pathological conditions. It is unequivocal that PTMs play important regulatory roles in protein functions, protein–protein/DNA interactions, and their stabilities, in which the study of disease-specific PTMs is instrumental for the development of potential biomarkers as well as targeted therapies [42,43,44]. One such broad spectrum dynamic PTM, acetylation, has been shown to govern a central role in BC and other cancers/diseases [8,11,27,37]. Heterogeneity in breast tumorigenesis is linked to epigenetic dysregulation and PTMs, involving enzymes and proteins, which affect the steroidogenic machinery and result in aberrant estrogen signaling [4,14]. Consequently, the key enzyme in estrogen synthesis, aromatase, invariably expressed in both cancerous and non-cancerous breast tissues, has been shown to be acetylated on several lysine residues in BC cells, in which the inhibition of SIRTs suppresses E2 biosynthesis but did not affect aromatase expression [8]. This indicates that E2 regulation in mammary tissue involves an alternate mechanism, and not solely due to aromatase-catalyzed events [10,11]. Concomitantly, the cholesterol transporter StAR is differentially expressed, along with E2 synthesis, in malignant and non-malignant breast tissues [11,20], representing StAR-driven E2 regulation in breast physiology and pathology. Moreover, upon analyses of The Cancer Genome Atlas Breast Invasive Carcinoma (TCGA-BRCA) RNA-Seq dataset, we reported that the amplification of the *StAR* gene, but not of *CYP19A1* or other steroidogenic enzyme genes such as cytochrome P450 family 11 subfamily A member 1 (*CYP11A1*), *CYP17A1*, 3*β*-hydroxysteroid dehydrogenase isoenzyme-1 (*HSD3B1*), or 17β-hydroxysteroid dehydrogenase (*HSD17B*), is correlated with poor survival of BC patients [4,15]. Our current data extend the observations by exemplifying the functional importance of uncovered StAR PTM, involving acetylation and deacetylation states at K111 and K253 positions, in diverse regulation of E2 synthesis in MCF7 and MB-231 BC cell models.

Aberrant and uncontrolled growth of mammary cells, promoting tumorigenesis, is influenced by genetic, epigenetic, and metabolic events, impacting DNA damage and genomic instability, immunodeficiency, and dysregulation of the epigenetic machinery [7,45,46,47]. Regardless of the alterations of molecular and cellular signaling, abundant expression of StAR, with corresponding E2 overproduction, is a critical event for breast pathogenesis [4,11,20]. It is well-established that the phosphorylation of StAR at Ser194/195 through cyclic AMP/protein kinase A signaling enhances the optimal cholesterol transporting capacity of StAR in steroid biosynthesis [48,49,50]. However, StAR was not found to be phosphorylated in hormone-sensitive BC in conjunction with elevated E2 levels [4,11], implying that StAR activity may change with other modifications or the involvement of unknown factors. In line with these findings, we uncovered a novel PTM and identified three lysine residues at K111, K238, and K253 in ER+ MCF7 cells that are intrinsically acetylated in the StAR protein, surmising that these StAR acetylated lysine residues contribute to E2 accumulation in hormone-sensitive BC. It is tempting to speculate that an aberrantly higher expression of StAR, involving acetylation, facilitates the increased delivery of cholesterol to the inner mitochondrial membrane and results in the overproduction of E2 for promoting breast tumorigenesis. Since cholesterol trafficking is also influenced by STARD3, a late endosomal START (StAR-related lipid transfer) domain protein with considerable homology to COOH-terminal StAR that was cloned initially in HER2+ BC [51,52], the potential modification(s) of STARD3, modulating E2 biosynthesis in breast tissue, cannot be excluded.

**Figure 4:**
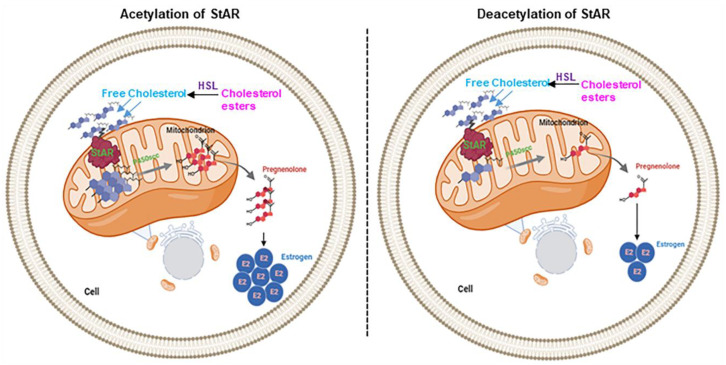
A schematic representation depicting the potential mechanisms associated with StAR acetylation and deacetylation events, involving cholesterol trafficking and utilization in mammary tissue. The availability of free cholesterol from cholesterol esters is an important event in steroid hormone biosynthesis, in which hormone-sensitive lipase (HSL) plays a key role. The StAR protein regulates steroid biosynthesis by controlling the intramitochondrial transport of cholesterol. The first steroid, pregnenolone, is formed at the mitochondria by the action of the cytochrome P450scc enzyme. Pregnenolone is then converted to various steroid hormones, including androgens and estrogen/E2, by tissue-specific enzymes. Acetylation of StAR increases its biological activity and results in the overproduction of E2 synthesis for promoting breast tumorigenesis (**left panel**). Alternatively, a StAR deacetylation event, involving the inefficient transport of cholesterol, suppresses E2 biosynthesis for preventing tumor growth and survival (**right panel**).

An intriguing aspect of the present findings is the functional relevance of StAR acetylation and deacetylation events to E2 biosynthesis in breast tissue. The identification of a total of fourteen lysine residues, along with the characterization of K111 and K253, involving K → Q and K → R mutants, to E2 regulation in mammary tissue opens up a new avenue in BC research and therapy. Importantly, StAR, by itself, was found to enhance E2 synthesis in BC cells, signifying that it could promote breast tumorigenesis and that it acts as a tumor promoter or oncogene. It should be noted that the inhibition of HDACs results in acetylation of not only histone and non-histone substrates, but also tumor suppressor proteins and oncogenes. As a consequence, HDACIs are capable of regulating a plethora of signaling processes, including cell cycle arrest, anti-proliferation, differentiation, apoptosis, and autophagy [4,11,35,36,37,38]. It is plausible that various StAR lysine residues, identified under basal- and HDACI-treated conditions, undergoing acetylation and deacetylation, have diverse effects on E2 synthesis in cancerous and non-cancerous breast tissues. In support of this, studies have demonstrated that the acetylation of mitochondrial proteins, such as StAR, exhibits both stimulatory and inhibitory effects on protein function and activity [53,54], resulting in both positive and negative effects on E2 levels in maintaining various breast functions. Histone modifications, including acetylation and deacetylation, are modulated by a balance between HATs and HDACs, and have been implicated in breast carcinogenesis, as well as its therapeutics [55,56,57]. Tumorigenesis involves genetic and epigenetic changes, which mediate tumor initiation, progression, and heterogeneity by disrupting the equilibrium between oncogenes and tumor suppressor genes [20,21,22]. An imbalance in the levels of HDACs (frequently overexpressed or mutated in malignant disorders) results in homeostatic disparity in the molecular networks that affect a variety of cellular and biological processes and modulate cancer etiology [30,52,58]. It is conceivable that, while the acetylation of StAR facilitates the accumulation of E2 for promoting breast tumorigenesis, the deacetylation of this cholesterol transporter suppresses E2 synthesis for preventing hormone-sensitive BC (Figure 4). Even so, StAR acetylation and deacetylation events and their correlation to E2 biosynthesis or other steroids may be context- and tissue-specific and involve discrete mechanisms, which require additional investigation. In pursuance of these data, we are currently generating antibodies to acetylated and deacetylated forms of StAR with K111Q, K253Q, K111R, and K253R, which could serve as molecular tools for BC screening/diagnosis and the regression of breast tumors, respectively. Moreover, an in-depth understanding of StAR acetylation to intra-tumoral E2 accumulation, and their correlation to HDACIs, is expected to provide mechanistic insights into breast pathogenesis along with clinical implications, leading to diagnostic, preventive, and therapeutic approaches for the most prevalent hormone-sensitive BCs.

The mechanism accounting for the suppression of intra-tumoral E2 accumulation, involving StAR expression and activity, is a fundamental event for combating hormone-sensitive BC [4,9,20]. It is unambiguous that the growth and survival of ER+ BC are activated by enhanced/accumulated E2 synthesis [20,59], in which the acetylation of StAR appears to play an indispensable role. Additionally, higher levels of cholesterol and its oxygenated derivatives, along with the *CYP27A1* enzyme, have been demonstrated to be instrumental in BC progression [60,61]. We recently reported that hormone-sensitive human breast tumors, as well as BC cell lines, possess higher expression of the StAR protein, compared with their normal counterparts or TNBCs [52]. Furthermore, analyses of the TCGA-BRCA RNA-Seq dataset, pertaining to largely luminal subtypes, identified the overexpression of several epigenetic enzymes compared with non-tumorous mammary epithelial cells [52]. Importantly, the dysregulation of the epigenetic machinery was found to be profoundly associated with the aberrant regulation of transcription factors that influence StAR activity, resulting in the overproduction of E2 for triggering breast tumorigenesis. Therefore, abnormality in genomic and epigenomic regulation, involving multiple factors/processes, impacts cholesterol metabolism and balance and results in E2 accumulation, in which StAR plays a key role. Accordingly, we reported that a variety of HDACIs, including three FDA-approved HDACIs, suppress E2 levels by altering StAR acetylation patterns, expression, and activity, not only in hormone-sensitive MCF7 cells, but also in primary cultures of enriched mouse breast tumor epithelial cells [8,11,20], emphasizing the potential of StAR as a therapeutic target for the management of ER+ BC. Moreover, the attenuation of StAR-governed E2 accumulation in BC cells/tissues by HDACIs could also be combined with other traditional therapies for improved BC treatment along with favorable patient outcomes. It is noteworthy that solid tumors, including breast, are inherently more genetically and epigenetically complex; thus, HDACI monotherapy targeting StAR, while ingenious, may not be entirely fruitful, due to off-target effects, drug resistance, and toxicity [62]. Therefore, combinatorial therapies of HDACIs with inhibitors of different molecular targets/signaling, including aromatase, proteasomes, epigenetic modifiers, immune molecules, and checkpoints, with less toxicity and higher tolerability, connecting the effective suppression of intra-tumoral E2 synthesis for hormone-sensitive BC or other relevant cancers, could be effective and beneficial for the prevention and/or treatment of this life-threatening disease of women globally.

The present study comprises some limitations, and the results should be interpreted prudently. Utilizing hormone-dependent and hormone-independent human breast cell lines, while a number of StAR acetylomes were identified, either endogenously or in response to various HDACIs, using LC-MS/MS, no acetylated StAR lysine residues were verified in primary breast tumors. Nevertheless, data generated with in vitro systems approach, even with a small sample size, substantially advance our understanding of E2 regulation in mammary tissue in the context of newly uncovered PTM of StAR, underlining that the notion of uncertainty is limited. It is noteworthy, however, that the iterative refinement of higher and lower E2 biosynthesis with two prominent K111/253 → Q and K111/253 → R mutants, respectively, in both MCF7 and MB-231 cells, provides not only mechanistic insights but also sheds light into clinical perspective. Additional studies, involving several other K → Q and K → R acetylation and deacetylation mutants, in pertinent cells and primary cultures of breast tumor epithelial cells, could evolve therapeutic implications targeting StAR in controlling intra-tumoral E2 accumulation for combating hormone-sensitive BC.

## 4. Materials and Methods

### 4.1. Culture of Cell Lines and Key Reagents

Cancerous breast cell lines were obtained from American Type Culture Collection (ATCC, Manassas, VA, USA), and were maintained in specific growth media containing antibiotics [8,11]. Specifically, hormone-dependent MCF7 (HTB-22, ATCC), hormone-independent MB-231 (HTB-26, ATCC), and non-cancerous mammary epithelial MCF12F (CRL-10783, ATCC) cell lines were used in this study. These cell lines were tested for mycoplasma contamination frequently and were utilized within 20 passages.

A number of key reagents were utilized in this study, as follows: Panobinostat (LBH589) was obtained from APExBIO (Houston, TX, USA); Vorinostat (SAHA) and SIRT 1 Inhibitors IV and VII were purchased from Millipore-Sigma (St. Louis, MO, USA); anti-StAR antibodies (Abs) (ab180804 or ab96637) were obtained from Abcam (Boston, MA, USA); and an ELISA kit for E2 was purchased from Cayman Chemical Co. (Ann Arbor, MI, USA) [8,11].

### 4.2. LC-MS/MS

The identification of StAR acetylated lysine residues in MCF12F, MCF7, and MB-231 cells was determined using LC-MS/MS (Thermo Fisher Ultimate 3000, Milford, MA, USA), following procedures described previously [8,11]. Briefly, these cells were cultured at 5 × 10^6^ cells per 150 mm cell culture dishes in regular growth media, 24 h prior to harvesting. In another set of experiments, MCF7 cells were washed with 0.01 M phosphate-buffered saline and treated without (DMSO), or with various HDACIs, i.e., Panobinostat (10 nM), SAHA (1 μM), IV (1 μM), or VII (1 μM) for 45min, under optimized conditions [11]. Following treatments, cells were collected and homogenized in radioimmunoprecipitation assay (RIPA) lysis buffer (50 mM Tris-HCl, pH 7.4, 150 mM NaCl, 1% Triton X-100, 0.5% NP-40, 10% glycerol, containing protease inhibitor cocktail (Thermo-Fisher Scientific, Waltham, MA, USA), 1 μM Trichostatin A and 1 mM nicotinamide, as described previously) [8,11]. Protein concentration in the cell lysate was quantified using the bicinchoninic acid assay method. Accordingly, 12–14 mg of total protein was incubated with 3–4 μg of StAR Ab (Abcam, Waltham, MA, USA) for 16 h at 4 °C on a Nutator. Protein–antibody complexes were then incubated with Protein G Dynabeads (Thermo-Fisher) for an additional 2 h [8,10,11]. Immune complexes were washed 4–6 times with lysis buffer and the final pellets were sent to Applied Biomics Inc. (Hayward, CA, USA) for identification of lysine residues that are acetylated in the StAR protein by LC-MS/MS. Acetylated StAR lysine residues depicting ion peaks at mass/charge (*m*/*z*) ratio of ~126 are summarized in Table 1 and Table 2.

### 4.3. Site-Directed Mutagenesis and Generation of StAR K → Q and K → R Acetylation and Deacetylation Mutants

Site-directed mutagenesis studies were employed to generate K → Q and K → R, acetylation and deacetylation mutants, using Quick-change II XL site-directed mutagenesis kit following the manufacturer’s protocol (Agilent Technologies, Santa Clara, CA, USA), as described previously [8,10]. Briefly, the pcDNA3-hStAR cDNA was subjected to mutagenesis with nucleotide primers selected using the Quick-change primer design tool (https://www.agilent.com/store/primerDesignProgram.jsp; assessed on 1 June 2023; Table 3). While acetylation mimetic mutants were generated by substitution of K → Q, deacetylation mutants were prepared with K → R, utilizing acetylated lysine residues at positions K111 and K253. The StAR plasmid was used as a double-strand DNA template in an 18-cycle polymerase chain reaction (PCR) with a reaction mix that consisted of 5 µL of 10× reaction buffer, 10 ng template, 125 ng oligonucleotide forward and reverse primers for each, 1 µL dNTP mix, 3 µL of Quick-change solution, made up to a final volume of 50 µL with ddH20, which includes 1 µL of *PfuUltra* HF DNA polymerase (2.5 U/µL). The initial denaturation was performed at 95 °C for 1 min; followed by 18 cycles of denaturation at 95 °C for 50 s, annealing at 60 °C for 50 s, and extension at 68 °C for 10 min; and a final extension at 68 °C for 7 min. Following the PCR reaction, 1 µL of *Dpn* I restriction enzyme (10 U/µL) was added to each amplification product and incubated at 37 °C for 1 h to digest the parental non-mutated supercoiled dsDNA. For PCR analyses, Veriti 96-well thermal cycler (Applied Biosystems; Foster City, CA, USA) was used [8,10]. The mutant plasmids were transformed into XL10 Gold ultra-competent cells (Agilent Technologies), screened bacterial colonies, and plasmids were purified [8,10]. All plasmids were confirmed by either restriction endonuclease digestion or sequencing on a PE Biosystem 310 Genetic Analyzer (Perkin-Elmer, Boston, MA, USA).

### 4.4. Transfection of WT-hStAR and, K → Q and K → R Mutants in MCF7 and MB-231 Cells

Transfection studies with these cells were carried out in 6-well plates with 1.5–2.0 μg of each DNA using Lipofectamine 3000 reagent (Invitrogen, Waltham, MA, USA), under optimized conditions [8,10,11,23]. Briefly, MCF7 and MB-231 cells were seeded at 2.5 × 10^5^ cells/well in 6-well plates in complete growth media. After 24 h of plating, cells were transiently transfected with either EV, WT-hStAR, or StAR containing K → Q and K → R acetylation and deacetylation mutants. Following 48 h of transfection, cells and culture media from different groups were collected and processed for appropriate analyses.

### 4.5. Immunoblotting

Immunoblotting analyses were performed using total cellular protein [8,10,11]. Briefly, MCF7 and MB-231 cells were extracted in RIPA lysis buffer containing protease inhibitor cocktail (Thermo-Fisher), centrifuged at 10,000× *g* for 10 min, and the supernatant was used for determining the total protein. Equal amounts of protein (60–75 µg) were loaded onto 10–12% SDS-PAGE (Bio-Rad Laboratories, Hercules, CA, USA) and transferred onto Immuno-Blot polyvineylidene fluoride membranes (Bio-Rad). Membranes were blocked with 5% milk in TBST (Tris-buffered saline containing 0.1% Tween 20) for 2 h at room temperature (RT), and probed with specific Abs that recognize StAR (ab96637, Abcam, 1:1000 dilution) and β-actin (PA5-141017; Ambion, Austin, TX, USA; 1:5000 dilution) for 16 h at 4 °C. The membranes were then washed and incubated with horseradish peroxidase-conjugated secondary Ab (ab97051, Abcam, 1:9000 dilution) for 1 h at RT. The immunodetection of StAR and β-Actin (assessed as a loading control) proteins was determined using a SuperSignal West Femto Chemiluminescence kit (Thermo Scientific), and analyzed using NIH ImageJ software (Version 1.43) [8,20,63].

### 4.6. Enzyme-Linked Immunosorbent Assay (ELISA)

E2 levels were assessed using an ELISA Kit from Cayman Chemical Co. (Ann Arbor, MI, USA), under optimized conditions [8,11]. Briefly, cell culture media, collected from different groups, were extracted with dichloromethane (4:1, *v*/*v*), snap frozen, and the top solvent layer was isolated. The solvent containing E2 was dried at either air or in a Savant speedVac vacuum concentrator (SPD130DLX, Thermo-Fisher), and resuspended in an ELISA buffer. E2 levels were expressed as ng/mg protein. The sensitivity of the E2 assay was 15 pg/mL, and the intra-assay coefficient of variation was less than 10%. Assays were performed in duplicates and absorbance was read at 412 nm on a Microplate Reader (Tecan; Mannedorf, Switzerland), as described previously [8,11]. 

### 4.7. Statistical Analysis

Statistical analyses were performed using a one-way analysis of variance (ANOVA) followed by a Tukey post hoc test using GraphPad Prism Software, version 9.5.1 (La Jolla, CA, USA), to assess whether differences observed in the experiments were significant. Student’s *t*-tests were used for analyzing significant differences between two groups. A *p*-value of less than 0.05 was considered statistically significant.

## 5. Conclusions

Estrogen signaling plays a central role not only in maintaining female reproductive development and function, but also in promoting breast tumorigenesis. How E2 elicits the symphony of two opposing activities, within the context of the indiscriminate expression of aromatase, an estrogen synthetase involving non-cancerous and cancerous breast tissues, remains puzzling. Alternatively, cholesterol is the precursor of all steroid hormones, including E2, in which the StAR protein predominantly regulates steroid biosynthesis; thus, it influences various cholesterol- and steroid-led physiological and pathophysiological activities. Along these lines, we reported that the StAR protein is abundantly expressed, concomitant with E2 synthesis, in hormone-sensitive human BC cells/tissues, compared with little to no StAR in either their non-cancerous counterparts or TNBC. The present findings, demonstrating the functional significance of StAR acetylation and deacetylation events to E2 biosynthesis, albeit limited, provide insights into the molecular regulatory switches that potentially impact intra-tumoral E2 accumulation, hence, breast tumorigenesis. Specifically, while StAR K111Q and K253Q acetylation mutants were found to increase the biological activity of StAR for the subsequent synthesis of E2, deacetylation mimetic events involving these lysine residues affected StAR and suppressed E2 buildup in BC cells, elucidating the relevance of StAR acetylation-based mechanistic events in E2 regulation in mammary tissue. Notably, a variety of FDA-approved and clinical phase trial HDACIs, at therapeutically and clinically relevant doses, have been shown to alter StAR acetylation patterns and subsequently suppress E2 accumulation in human and mouse ER+ BC cells/tissues [11,20]. Taken together, the results of the present findings, provide molecular insights into E2 regulation impacting breast physiology and pathology by typifying the following events: (i) StAR protein is acetylated at several lysine residues in cancerous and non-cancerous breast cells, (ii) the identification of this novel StAR PTM in human adrenal cells underscores its importance in steroid hormone regulation and function, (iii) the overexpression of StAR enhances E2 levels in BC cells implying that StAR promotes breast tumorigenesis, (iv) the acetylation of StAR is an important event in intra-tumoral E2 accumulation, (v) HDACIs alter StAR acetylation patterns and suppress E2 synthesis in ER+ BC cells, and (vi) the acetylation and deacetylation of StAR mutants at K111 and K253 exhibit opposing effects on E2 biosynthesis in BC cells.

## Figures and Tables

**Table 1:** Identification of StAR lysine residues (highlighted in red) that are putatively acetylated endogenously in MCF12F, MCF7, and MB-231 cells using LC-MS/MS.

Lysine Residues	MCF12F (Normal)	MCF7 (ER+/PR+)	MB-231 (TNBC)	Representative Peptide
K7			+	MLLATFK
K97	+			ALGILSNQEGWKK
K98	+		+	KESQQDNGDK
K111		+		ESQQDNGDKVMSK
K118			+	VVPDVGKVFR
K155	+			MEAMGEWNPNVKEIK
K238		+		AEHGPTCMVLHPLAGSPSKTK
K253		+	+	LTWLLSIDLKGWLPK

**Table 2:** Acetylated StAR lysine residues (highlighted in red) under basal (DMSO, dimethyl sulfoxide) and Panobinostat (PANO)-, SAHA-, IV-, and VII-treated conditions in MCF7 cells.

Lysine Residues	MCF7 (ER+/PR+)					Representative Peptide
	Basal	PANO	SAHA	IV	VII	
K21				+		HMRNMK
K107		+		+	+	KESQQDNGDK
K111	+			+	+	ESQQDNGDKVMSK
K118			+			VVPDVGKVFR
K152		+	+			LYEELVERMEAMGEWNPNVK
K162		+	+	+	+	VLQKIGK
K213				+	+	GSTCVLAGMATDFGNMPEQKGVIR
K238	+					AEHGPTCMVLHPLAGSPSKTK
K248			+			TKLTWLLSIDLK
K253	+	+	+	+	+	LTWLLSIDLKGWLPK

**Table 3:** Site-directed mutagenesis primer pairs were used for generating StAR K → Q and K → R acetylation and deacetylation mutants at positions K111 and K253.

Mutants	Forward Primer	Reverse Primer
K111Q	5′-cccacatctgggaccacctgactcatcactttgtccc-3′	5′-gggacaaagtgatgagtcaggtggtcccagatgtggg-3′
K111R	5′-cacatctgggaccactctactcatcactttgtccc-3′	5′-gggacaaagtgatgagtagagtggtcccagatgtg-3′
K253Q	5′-gttgatgatgctctggggcagccacccct-3′	5′-aggggtggctgccccagagcatcatcaac-3′
K253Q	5′-ggttgatgatgctcctgggcagccacccc-3′	5′-ggggtggctgcccaggagcatcatcaacc-3′

## Data Availability

The original contributions presented in the study are included in the article, further inquiries can be directed to the corresponding author.
